# A Novel Approach for In Vitro Testing and Hazard Evaluation of Nanoformulated RyR2-Targeting siRNA Drugs Using Human PBMCs

**DOI:** 10.3390/life15010095

**Published:** 2025-01-14

**Authors:** Valeria Bettinsoli, Gloria Melzi, Angelica Crea, Lorenzo Degli Esposti, Michele Iafisco, Daniele Catalucci, Paolo Ciana, Emanuela Corsini

**Affiliations:** 1 Laboratory of Toxicology and Risk Assessment, Department of Pharmacological and Biomolecular Sciences “Rodolfo Paoletti”, Università degli Studi di Milano, 20133 Milan, Italy; valeria.bettinsoli@unimi.it (V.B.); emanuela.corsini@unimi.it (E.C.); 2 Department of Pharmacy, Università degli Studi di Napoli Federico II, 80131 Naples, Italy; 3 Dipartimento di Chimica e Chimica Industriale, Università degli Studi di Genova, 16146 Genoa, Italy; lorenzo.degliesposti@unige.it; 4 Institute of Science, Technology and Sustainability for Ceramics (ISSMC), National Research Council (CNR), 48018 Faenza, Italy; michele.iafisco@issmc.cnr.it; 5 Institute of Genetic and Biomedical Research (IRGB), National Research Council (CNR), 20133 Milan, Italy; daniele.catalucci@cnr.it; 6 IRCCS Humanitas Research Hospital, 20089 Rozzano, Italy; 7 Department of Health Sciences, Università degli Studi di Milano, 20146 Milan, Italy; paolo.ciana@unimi.it

**Keywords:** new approach methodologies, peripheral blood mononuclear cells, nucleic acid drugs, immunotoxicology, toxicity pathways, RyR2, nanoparticles

## Abstract

Nucleic acid (NA)-based drugs are promising therapeutics agents. Beyond efficacy, addressing safety concerns—particularly those specific to this class of drugs—is crucial. Here, we propose an in vitro approach to screen for potential adverse off-target effects of NA-based drugs. Human peripheral blood mononuclear cells (PBMCs), purified from buffy coats of healthy donors, were used to investigate the ability of NA-drugs to trigger toxicity pathways and inappropriate immune stimulation. PBMCs were selected for their ability to represent potential human responses, given their likelihood of interacting with administered drugs. As proof of concept, a small interfering RNA (siRNA) targeting Ryanodine Receptor mRNA (RyR2) identified by the Italian National Center for Gene Therapy and Drugs based on RNA Technology as a potential therapeutic target for dominant catecholaminergic polymorphic ventricular tachycardia, was selected. This compound and its scramble were formulated within a calcium phosphate nanoparticle-based delivery system. Positive controls for four toxicity pathways were identified through literature review, each associated with a specific type of cellular stress: oxidative stress (tert-butyl hydroperoxide), mitochondrial stress (rotenone), endoplasmic reticulum stress (thapsigargin), and autophagy (rapamycin). These controls were used to define specific mRNA signatures triggered in PBMCs, which were subsequently used as indicators of off-target effects. To assess immune activation, the release of pro-inflammatory cytokines (interleukin-6, interleukin-8, tumor necrosis factor-α, and interferon-γ) was measured 24 h after exposure. The proposed approach provides a rapid and effective screening method for identifying potential unintended effects in a relevant human model, which also allows to address gender effects and variability in responses.

## 1. Introduction

In recent years, nucleic acid (NA)-based drugs have emerged as a promising class of therapeutics. These molecules modulate the expression of specific RNA transcripts by leveraging natural cellular mechanisms [1]. This technology allows the generation of a specific sequence complementary to target RNAs, leading to the modulation of their expression [2]. This strategy not only provides precise control over gene expression but also allows for temporary modulation, making it a powerful tool in gene regulation and therapeutic development. The specificity of these molecules reduces the likelihood of side effects, making RNA drugs promising candidates for leading the next wave of precision medicine [3,4].

RNA-based therapies have made significant progress, with several achieving clinical approval and others in investigational or preclinical stages. Various classes of antisense molecules, including small interfering RNAs (siRNAs), have been evaluated in clinical trials for conditions including glaucoma [5], dry eye syndrome [6], diabetic macular oedema [7], age-related macular degeneration [7], amyloidosis [8], dyslipidemia [9,10], and hypertension [11,12]. Although the development of RNA-based therapeutics faces considerable challenges, multiple strategies—such as enhancing intracellular RNA trafficking and improving metabolic stability—have been explored to overcome these hurdles [13]. RNA therapies are particularly promising for modifying gene expression or producing therapeutic proteins, making them ideal for diseases with well-defined genetic targets. They hold significant potential for treating infectious diseases, cancers, immune disorders, Mendelian conditions, and various neurological disorders [14].

In the field of siRNA therapy, current efforts focus on optimizing this technology to achieve effective gene silencing while minimizing its adverse in vivo effects. The therapeutic potential of RNA interference has driven scientists to develop modified siRNA molecules that enhance gene silencing efficiency, enabling the full exploitation of this powerful mechanism [15].

Evaluating the safety of RNA-based drugs involves multiple factors. The drug’s ability to specifically interact with its target is essential for achieving selective therapeutic effects while avoiding unintended off-target side effects. This is particularly relevant for exogenous RNA, which can trigger both sequence-dependent and sequence-independent responses [16]. On-target toxicities, stemming from exaggerated pharmacological effects, are influenced by the RNA sequence and its hybridization properties. Such toxicities may occur when the therapeutic response is excessively strong or when harmful effects manifest in non-targeted organs. Additionally, off-target risks related to Watson–Crick base pairing arise when the RNA inadvertently affects non-target transcripts [17]. The effectiveness of RNA therapies also relies on successful delivery into the cytoplasm, which requires overcoming multiple extracellular and intracellular barriers [13].

Immune-related toxicities and dose-limiting adverse effects are major contributors to the failure of RNA drugs in clinical trials [18]. These toxicities often involve interactions with Toll-like receptors (TLR) in cell membranes or endosomes, as well as cytosolic sensors such as RNA-dependent protein kinase [19]. When immune receptors recognize specific RNA sequences or structures, they can trigger responses such as cytokine secretion, immune cell activation, and the initiation of adaptive immunity. Consequently, RNA-based therapeutics may induce inappropriate immunostimulation with the induction of cytokine release syndrome; influenced by dose and structural characteristics such as RNA size and sequence [20]. Selectivity and safety also depend on dosage, as higher doses of RNA interference agents can lead to off-target effects that limit their use [21]. Thus, designing optimal RNA sequences, incorporating chemical or natural modifications to reduce immunogenicity and off-target risks, and determining a dose that balances efficacy and safety are all critical steps for successful RNA drug development [16].

Another aspect to consider when developing RNA drugs is the delivery system to use to protect the delicate structure of NA therapeutics and allow specific targeting. In fact, RNAs are highly susceptible to destruction by enzymes such as nucleases or hydrolases in blood or body fluids, and rapidly eliminated by the kidney [22]. To overcome these issues, RNA therapies typically use specific carriers for delivery. It is vital to assess the safety of both the carrier materials and the final drug product. Issues such as toxicity from carrier degradation during storage, infusion site reactions, or unintended accumulation in non-target tissues must be carefully evaluated [23]. Many clinical trials of RNA-based drugs and vaccines have stalled at phase I or II due to low efficacy or unexpected shifts in the risk–benefit profile. Preclinical safety assessments aim to identify formulations that are both effective and well tolerated. In vitro and ex vivo tests play a crucial role in evaluating the pharmacological activity of drug candidates and refining them based on cytotoxic effects and impacts on cell survival. These human cell-based assays provide a cost-effective and practical approach to addressing species-specific differences in immune and genomic responses [24].

This study aimed to evaluate the safety of a nanoformulated siRNA developed to target the mutated cardiac Ryanodine Receptor gene (RyR2) that causes dominant catecholaminergic polymorphic ventricular tachycardia (CPVT). The cardiac gene RyR2 is responsible for the calcium release from sarcoplasmic reticulum (SR) inducing muscle contraction; mutation related to this gene can induce uncontrolled muscle contraction, causing arrythmias that can lead to cardiac arrest [25]. Syncopal attack triggered by exercise is one of the representations of the many manifestations of CPVT in the pediatric population [26]. The gene RyR2 encodes a protein that forms a homotetrameric ion channel localized in the membrane of the junctional sarcoplasmic reticulum. RyR2 channels are aligned with L-type Ca^2+^; channels (LTCC) that are located within specialized invaginations of the cellular membrane, termed transverse tubules. The influx of Ca^2+^; through LTCCs induces RyR2-mediated Ca^2+^; release from the SR; a process known as calcium-induced calcium release, which facilitates cardiac muscle contraction [27]. Under normal conditions, sympathetic activation can induce phosphorylation of RyR2, further enhancing Ca^2+^; release from the SR in myocytes. However, in the presence of mutations, there is an improper release of calcium ions from the SR, leading to irregular heartbeats. This effect is further aggravated by adrenergic activation [28].

SiRNA RyR2-U10 has been developed in the context of the Italian National Center for Gene Therapy and Drugs based on RNA Technology. It can recognize the mutated sequence RyR2 in position RYR2-R4496C while sparing the wild-type form [29]. Nanoparticles (NPs) were used as a siRNA delivery platform to provide an efficient drug delivery strategy towards the cardiac site. RyR2-R4496C targeting siRNA has been loaded into negatively charged calcium phosphate (CaP) NPs using a biomineralization-inspired approach [30,31]. These biocompatible nanocarriers, with sizes ranging from 50 to 100 nm, are stable in physiological conditions and enable safe and effective delivery into cardiac tissue [30]. Inhalation was selected as the delivery route for administering the biomolecule-loaded CaP NPs to cardiomyocytes [31,32,33]. In fact, this unconventional administration method for targeting the heart is based on findings that demonstrate the presence of combustion-derived NPs and ultrafine particulates in the heart following inhalation of polluted air. Upon trans crossing the thin alveolar epithelium, the particulate fast reaches the myocardium via the pulmonary circulation that carries the oxygenated blood from the lungs to the heart [34,35]. In vivo testing demonstrated that inhalable CaP NPs loaded with therapeutic biomolecules can effectively translocate from the lungs to the heart, penetrate cardiomyocytes via endocytosis, and release their payload through acidic dissolution in endosomes (endosomal escape); thereby treating cardiovascular disease [31,33,36].

This study aimed to develop a screening method to evaluate the potential toxicity of RNA-based drugs. The safety evaluation was completed in vitro using peripheral blood mononuclear cells (PBMCs). These cells were treated with the CaP-loaded RNA-drug and the endpoints evaluated included cytotoxic effect, inappropriate immune response, and activation of toxicity pathways (autophagy, endoplasmic reticulum stress, mitochondrial stress, and oxidative stress). Data were collected following 24 h of treatment with the drug at therapeutic dosage or higher concentrations to stress the system and detect possible adverse effects. Protocols were standardized and SOPs are available upon request.

This method serves as a preliminary step in the preclinical safety assessment of RNA-based therapeutics.

## 2. Materials and Methods

### 2.1. Materials

Calcium chloride, sodium citrate, sodium hydroxide, sodium hydrogen phosphate, Dulbecco’s Phosphate Buffered Saline, RPMI-1640, Gentamicin, human serum, tert-Butyl Hyperoxide, Thapsigargin, Rotenone, Rapamycin, and dimethyl sulfoxide were purchased by Sigma Aldrich (St. Louis, MO, USA). siRNA RyR2 sense strand: UAUUUUGCUUGCAACUUUUAC[dT][dT], antisense strand: GUAAAAGUUGCAAGCAAAAUA[dT][dT]; siRNA scramble: MISSION^®^ siRNA Universal Neg. Controls were purchased by Merck (Darmstadt, Germany). RNAse-free ultrapure water, Quant-it microRNA assay kit, and Fluoroskan Microplate Fluorometer were purchased by Thermo Fisher Scientific (Waltham, MA, USA). L-Glutamine, 0.1 mg/mL streptomycin, 100 U/mL penicillin, CyQUANT™ LDH Cytotoxicity Assay Kit were purchased from Invitrogen™ by Thermo Fisher Scientific (Monza, Italy). Zetasizer Nano ZSP instrument was purchased by Malvern Instruments (Malvern, UK). Laser Doppler velocimetry DTS1061 was purchased by Malvern Ltd. (Worcestershire, UK). Lympholyte^®^ Cell Separation Media was purchased by Cedarlane, EuroClone (Pero, Milan, Italy). 2-mercaptoethanol was purchased by Bio-Rad Laboratories (Segrate, Milan, Italy). Spectrophotometer Emax was purchased by Molecular Devices (San Jose, CA, USA). ELISA kits were purchased by Bio-Techne (IL-6, IFN-γ, and TNFα; Minneapolis, MN, USA), and ImmunoTools (IL-8; Friesoythe, Germany). RNA-Zol Direct Clean-Up Plus Kit was purchased by Fisher Molecular Biology (Rome, Italy). QuantiTect Reverse Transcription Kit, QuantiNova™ SYBR^®^ Green PCR Kit, and QuantiTect Primer Assays were purchased by Qiagen (Milan, Italy). Spectrum Spectra Por 7 cellulose membrane (MWCO: 3500 Da) was purchased by Spectrum labs (Repligen) Waltham, MA, USA.

### 2.2. Synthesis of siRNA Loaded CaP NPs

CaP NPs functionalized with either scramble or RyR2 siRNAs (CaP-scramble and CaP-siRNA, respectively) were synthesized using a modified version of a previously reported method [30,31]. Specifically, CaP-siRNA NPs were generated by mixing two aqueous solutions (1:1 *v*/*v*; 20 mL total) of (A) 0.1 M calcium chloride (CaCl_2_∙2 H_2_O, ≥99.0% pure) + 0.4 M sodium citrate (Na_3_(C_6_H_5_O_7_)∙2H_2_O, ≥99.0% pure) + 7.14 mM sodium hydroxide (NaOH, ≥99.0% pure) and (B) 0.12 M sodium hydrogen phosphate (Na_2_HPO_4_, ≥99.0% pure) + 4 µM siRNA (either scramble or active, ≥99.0% pure). The precipitation was induced by keeping the mixture under vigorous stirring for 5 min at 37 °C in a water bath. Afterwards, siRNA-CaP NPs were purified by dialysis using a Spectrum Spectra Por 7 cellulose membrane (MWCO: 3500 Da) (Spectrum labs (Repligen) Waltham, MA, USA) for 24 h against ultrapure water with multiple water exchanges. CaP-siRNA NPs suspension was stored at 4 °C until further use. Unloaded CaP NPs were prepared as reported above, but without adding siRNA in solution (B). RNAse-free ultrapure water was used for all preparations. All reagents were used without further purification.

### 2.3. Characterization of siRNA Loaded CaP NPs

Quantification of siRNA was performed by using the Quant-it microRNA assay kit according to the manufacturer’s instructions. Calibration curves of scramble and active siRNA between 0 and 2 µM were used. Fluorescence intensity was measured with a Fluoroskan Microplate Fluorometer at excitation and emission wavelengths of 485 and 538 nm, respectively. Three replicates were performed for each sample. siRNA payload is expressed as a weight percentage (wt.%) relative to the mass of CaP NPs. siRNA-CaP NPs hydrodynamic diameter distribution and surface charge in native suspension were analyzed through dynamic light scattering (DLS) and electrophoretic mobility (ζ-potential), respectively, by using a Zetasizer Nano ZSP instrument. Nanoparticle size, reported as the Z-average of the hydrodynamic diameter distribution, represents the mean of three measurements at 25 °C of at least 10 runs. Measurements were calculated using the hydroxyapatite refractive index (1.63) for the samples, and water’s viscosity (0.8872 cP) and refractive index (1.33) as solvent parameters. The ζ-potential was quantified as the electrophoretic mobility at 25 °C of three separate measurements (maximum 100 runs each) by laser Doppler velocimetry using a disposable electrophoretic cell using the same sample and solvent parameters.

### 2.4. PBMCs Isolation

Buffy coats from healthy anonymous donors, female and male, were purchased from the Niguarda Hospital in Milan (Italy). PBMCs were obtained by Ficoll gradient centrifugation using Lympholyte^®^ Cell Separation Media. After centrifugation, PBMCs layer was removed and washed 5 times with Dulbecco’s Phosphate Buffered Saline (PBS). Isolated cells were diluted to 10^6^ cells/mL in RPMI-1640 medium supplemented with 2 mM L-glutamine (CAS#56-85-9), 0.1 mg/mL streptomycin, 100 U/mL penicillin, 0.1% gentamicin, and 0.1% 2-mercaptoethanol (CAS#60-24-2) and 5% heated-inactivated human serum and cultured at 37 °C in a humidified atmosphere of a 5% CO_2_ incubator. PBMCs were seeded at the cell concentration of 10^6^ cells/mL in 24-multiwell, 1 mL for each well.

### 2.5. Treatment with Positive Controls, RyR2 CaP-siRNA and CaP-Scramble Drugs

Literature research allows the selection of positive controls for the four toxicity pathways: oxidative stress, mitochondrial stress, endoplasmic reticulum stress, and autophagy. The compounds that have been selected are tert-Butyl Hyperoxide (tBHP), Thapsigargin (TG), Rotenone (Rot), and Rapamycin (Rapa), respectively.

PBMCs were treated with positive controls, CaP-siRNA, and CaP-scramble for 24 h. The treatment with positive controls was performed with tBHP, TG, Rot, and Rapa at the concentration resulting in 80% of cell viability (CV80); 3.125 µM for tBHP, 2 µM for TG, and 50 µM for Rot and Rapa. The substances under analysis were reconstituted in DMSO, which was therefore used as vehicle control treatment (0.1% DMSO as final concentration). For the treatment with RNA-drugs, PBMCs were exposed to increasing concentrations of CaP loaded with RyR2 siRNA, CaP loaded with siRNA-scramble, and unloaded CaP NPs at three different siRNA concentrations: 100 nM, 200 nM, and 400 nM. For each concentration, the relative amount of CaP NP is respectively: 0.0315 mg/mL for 100 nM, 0.063 mg/mL for 200 nM, and 0.126 mg/mL for 400 nM.

**Figure 1:**
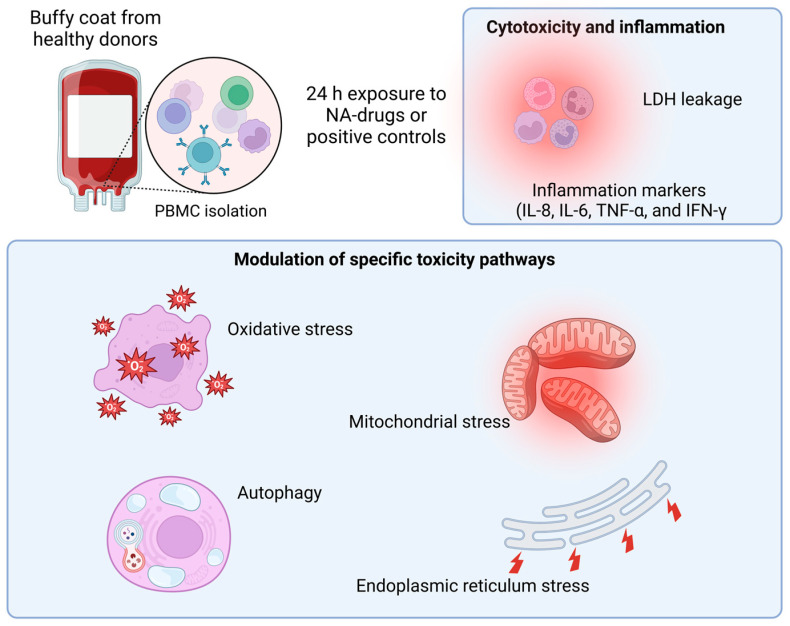
Experimental design of the study; created in BioRender. https://BioRender.com/s74d404 (accessed on 14 December 2024). PBMC: peripheral blood mononuclear cells; NA-drugs: nucleic-acid drugs; LDH: lactate dehydrogenase; IL: interleukin; TNF: tumor necrosis factor; IFN: interferon.

Unloaded CaP NPs were used as control. The experimental design is reported in Figure 1.

### 2.6. Cytotoxicity Evaluation

Cytotoxicity was assessed at the end of treatment by measuring the leakage of lactate dehydrogenase (LDH). The concentration resulting in 80% cell viability (CV80) was calculated by linear regression analysis of data.

The measurement of LDH leakage was performed using the CyQUANT™ LDH Cytotoxicity Assay Kit for both siRNA drugs and positive controls. After the treatment, plates were centrifuged for 5 min at 1500 rpm; following which, 50 μL of supernatant from each sample were transferred to a new 96-well plate, following the manufacturer’s instructions. A total of 50 μL of the reaction mix solution were added in each well and the plate was incubated at the dark for 30 min when the stop solution was added. Absorbance was measured at 680 nm and 490 nm using a spectrophotometer.

### 2.7. Cytokines Release

PBMC were treated with positive controls or siRNA nano-formulations for 24 h. Cells were centrifuged at 1500 rpm for 8 min and supernatants collected for cytokine measurement and stored at −20 °C until measurement. Cytokine production was assessed in cell-free supernatants by specific sandwich enzyme-linked immunosorbent assays (ELISA) that are commercially available. Results were expressed as fold-change of released cytokines of treated samples versus control cells. Sensitivity range: IL-6 9.38–600 pg/mL; IFN-γ 9.38–600 pg/mL; TNFα 15.6–1000 pg/mL; IL-8 8–500 pg/mL.

### 2.8. Toxicity Pathways Evaluation

The activation of the toxicity pathways was performed by analyzing the expression of specific genes involved in autophagy, endoplasmic reticulum stress, mitochondrial stress, and oxidative stress. The genes were selected following bibliographic research, with confirmed expression in PBMCs after treatment with the positive controls, and are reported in Table 1. The expression of these genes was evaluated using real-time PCR (RT-PCR). mRNA was extracted following the instructions provided by the RNA-Zol Direct Clean-Up Plus Kit, and then retro-transcribed with the QuantiTect Reverse Transcription Kit. A quantity of 6 ng of cDNA was used. RT-PCR was performed with QuantiNova™ SYBR^®^ Green PCR Kit and QuantiTect Primer Assays. RNA 18S subunit was used as a housekeeping gene.

### 2.9. Statistical Analysis

Results are reported as mean ± standard deviation (SD) of 7 donors (5 males and 2 females). Statistical analysis was performed using GraphPad Prism 8.0.2 (GraphPad Software, San Diego, CA, USA). Outliers were identified following the statistical test and removed from the mean. A one-way ANOVA test was chosen for the analysis of all results, in association with Dunnett’s Multiple Comparison post hoc test. Results were considered significant at *p* < 0.05.

## 3. Results

### 3.1. Chemical-Physical Properties of siRNA-Loaded CaP NPs

CaP NPs loaded with RyR2 siRNA (CaP-siRNA), scramble siRNA (CaP-scramble), and non-loaded CaP NPs were characterized in terms of NP concentration, siRNA payload, size, and surface charge (Table 2). All samples were comparable in terms of particle size and surface charge, with a hydrodynamic diameter of approximately 40–60 nm and a strongly negative surface charge (−30/−35 mV). siRNA-loaded CaP NPs exhibited a slightly smaller hydrodynamic diameter compared to non-loaded CaP NPs, suggesting that siRNA acts as a stabilizer during synthesis by associating with forming nuclei and limiting particle growth. NP concentrations were consistent across all samples; however, the siRNA payload was higher for RyR2 siRNA than for scramble siRNA, indicating a stronger association with CaP NPs. This observation aligns with the smaller hydrodynamic diameter observed for CaP-siRNA.

### 3.2. Cytotoxic Effects

To investigate the in vitro effects of the selected compounds, cell viability was first assessed to identify the maximum viable concentrations (CV80). PBMCs were exposed to increasing concentrations (100, 200, 400 nM) of CaP-siRNA and CaP-scramble where the lowest concentration tested (100 nM) is close to what is currently considered the in vitro therapeutic dose. Empty CaP were not cytotoxic at the concentrations tested [29].

**Figure 2:**
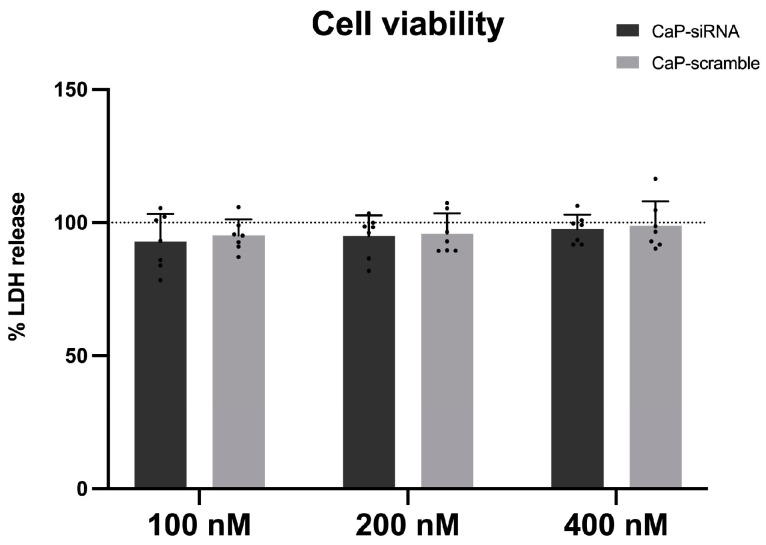
Effect of CaP-siRNA and CaP-scramble on cell viability. PBMCs were treated with CaP NPs loaded with RyR2 siRNA (CaP-siRNA), scramble siRNA (CaP-sramble), and non-loaded CaP NPs at three different concentrations (100, 200, 400 nM) for 24 h. The results are reported as percentage of LDH release normalized on CaP NPs (dashed line). Each column represents the mean ± SD (n = 7).

The results obtained (Figure 2) indicate no cytotoxic effects induced by CaP-siRNA and CaP-scramble at all the concentrations tested, compared to the control (CaP NPs), which were previously proved to be non-cytotoxic [30,31].

### 3.3. Cytokines Release

**Figure 3:**
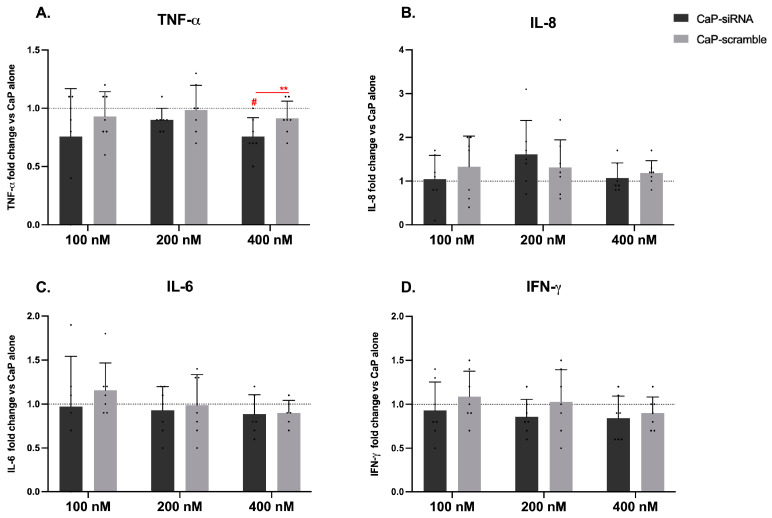
Effect of CaP-siRNA and CaP-scramble on cytokines release. PBMCs were exposed to CaP NPs loaded with RyR2 siRNA (CaP-siRNA), scramble siRNA (CaP-scramble), and non-loaded CaP NPs at three different concentrations (100, 200, 400 nM) for 24 h. TNF-α (**A**), IL-8 (**B**), IL-6 (**C**), and IFN-γ (**D**) release were assessed by specific ELISA. Results are normalized on the cytokine release in CaP NPs-treated cells (dashed line). Each column represents the mean ± SD (*n* = 7). Statistical analysis was performed by one Way ANOVA, Dunnett’s Multiple Comparison test, with # *p* < 0.05 vs. CaP NPs and ** *p* < 0.01 vs. CaP-scramble.

In Figure 3, the effect of CaP-loaded RyR2 siRNA and scramble siRNA, as well as non-loaded CaP NPs on cytokine release is reported. Results are expressed as changes over CaP-induced release.

No changes in IL-8, IL-6, and IFN-γ releases were observed after CaP-siRNA or, CaP-scramble (Figure 3B–D). TNF-α levels were statistically significantly reduced after 24 h (Figure 3A) in CaP-siRNA treated cells at the concentration of 400 nM compared to both non-loaded CaP NPs (#) and CaP-scramble (**) groups.

### 3.4. Effects on Toxicity Pathways

**Figure 4:**
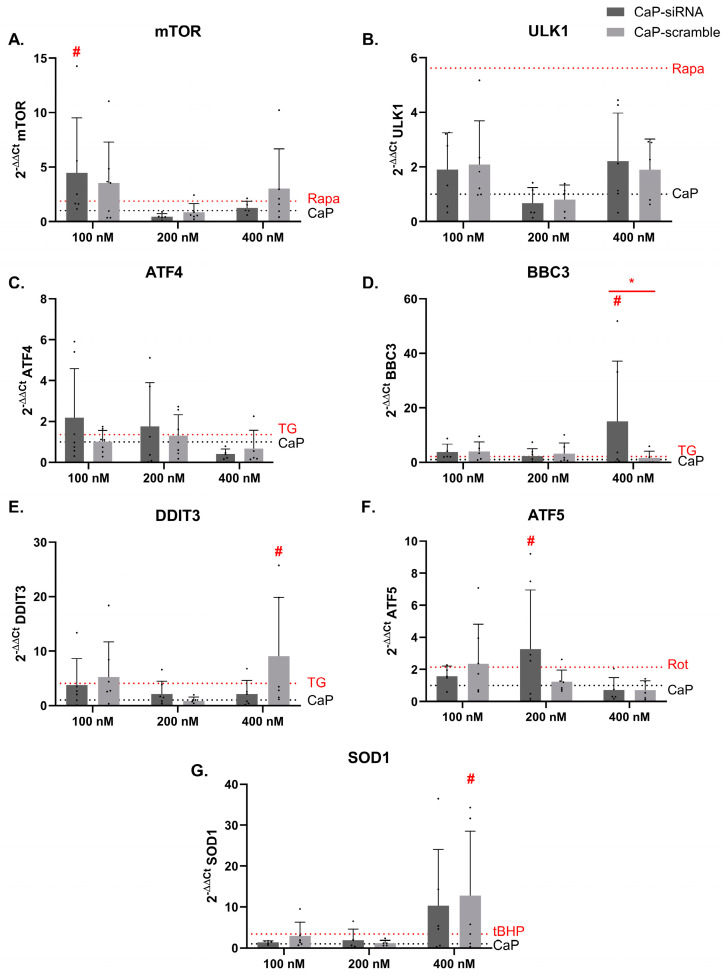
Modulation of the genes involved in the toxicity pathways. The expression of the selected genes was analyzed by RT-PCR 24 h after exposure to CaP NPs loaded with RyR2 siRNA (CaP-siRNA), the scramble siRNA (CaP-scramble), or non-loaded CaP NPs at three different concentrations (100, 200, 400 nM). Results are reported as 2^−ΔΔct^ and normalized on the non-loaded CaP NPs gene expression. In the graphs, the results of the gene modulation are reported: autophagy (**A**) mTOR and (**B**) ULK1; ER stress (**C**) ATF4, (**D**) BBC3, and (**E**) DDIT3; mitochondrial stress (**F**) ATF5; oxidative stress (**G**) SOD1. The black dashed line represents the CaP NPs, and the red dashed line represents the positive controls selected for each pathway (Rapa, TG, Rot, or tBHP). The data of the positive controls are normalized on the DMSO vehicle. Each column represents the mean ± SD, (*n* = 7). Statistical analysis: One Way ANOVA, Dunnett’s Multiple Comparison test. # *p* < 0.05 vs. CaP NPs; * *p* < 0.05 vs. CaP-scramble.

The activation of the toxicity pathways was analyzed by RT-PCR following 24 h of treatments with the CaP-siRNA, CaP-scramble, and non-loaded CaP NPs at the concentrations 100, 200, and 400 nM, and positive controls. The results shown in Figure 4 are related to modulation of the mRNA levels of genes involved in autophagy (A) mTOR and (B) ULK1; ER stress (C) ATF4, (D) BBC3, and (E) DDIT3; mitochondrial stress (F) ATF5; and oxidative stress (G) SOD1.

A statistically significant increase in mTOR mRNA was detected with 100 nM CaP-siRNA. This effect was not dose-related and was evident only at the lowest concentration. Regarding the genes involved in ER stress, there was a statistically significant increase of the expression of BBC3 mRNA expression following treatment with 400 nM CaP-siRN; and of DDIT3 mRNA following treatment with 400 nM CaP-scramble. Moreover, a statistically significant difference in BBC3 mRNA expression was observed between CaP-siRNA and CaP-scramble at a concentration of 400 nM. The gene involved in mitochondrial stress, ATF5, was significantly modulated following treatment with 200 nM CaP-siRNA. SOD1, the gene involved in the oxidative stress response, showed a statistically significant increase in mRNA expression following treatment with 400 nM CaP-scramble.

Generally, no clear concentration-dependent effects were observed; with induction values similar or higher to the ones induced by the positive controls (Rapa, TG, Rot, and tBHP). Additional studies are needed to better substantiate the effects and possible consequences.

## 4. Discussion

This study aimed to establish a screening method to explore the potential toxicity of RNA-based drugs. CaP-siRNA and CaP-scramble, formulated in CaP nanoparticles, were used as proof-of-concept reference compounds. The proposed approach addressed both immunotoxicity by examining the release of proinflammatory cytokines, and the activation of toxicity pathways through the analysis of key gene expressions. These analyses were conducted as a preliminary step in the preclinical assessment of the safety profile of RNA-based drugs.

The proposed model is relevant because it uses human cells, allowing for the investigation of possible gender effects and variability in responses. Genetic and/or epigenetic differences may influence individual sensitivity to drug effects and toxicity, reducing the consistency of the data. However, the results indicate the possibility that some people may be more sensitive than others, which requires additional investigations.

The strategy proposed in this study takes advantage of the use of PBMCs, as an in vitro model, which is relevant to translate results into the human organism.

The siRNA and the scramble evaluated in this study are loaded into negatively charged CaP NPs; an efficient tool that allows the internalization of the drug in the cells. The amount of RNA loaded into CaP NPs (approximately 2–3 wt.%) has previously demonstrated therapeutic activity in cardiomyocytes without causing toxicity or interfering with any functional properties [29,31]. This innovative system is designed for targeted drug delivery to the heart via inhalation [30,44,45]. siRNA loading does not alter significantly the physico-chemical characteristics of CaPNPs; as polydispersity, surface charge, and concentration remain comparable between unloaded and siRNA-loaded CaP NPs. However, NPs with siRNA exhibit a slightly smaller size; likely due to a stabilization effect imparted by the siRNA.

When developing NPs as drug delivery vectors, a key consideration is their presence in the circulatory system. Upon entering the bloodstream, NPs are often recognized as foreign, prompting immune cells to target them for removal [46]. Another challenge is represented by the potential activation of toxic reactions and other incompatibilities between the NPs and the blood environment. The investigation of these aspects is crucial to ensure safe and effective delivery [47]. Indeed, it is essential during drug development to use appropriate in vitro and in vivo models to accurately analyze the potency and toxicity of these molecules. Depending on the model used, different reactions can be observed; both to the RNAs and to the excipients used. The results of this study showed the absence of activation of the toxicity pathway analyzed following treatment with the CaP NPs—either unloaded or loaded with siRNAs—proving their safety as RNA delivery vectors.

Today, there are several RNA drugs on the market; including, but not limited to, mRNA vaccines [13]. Some siRNA drugs have also been commercialized (i.e., inclisiran for contrasting dyslipidemia), as well as certain ASOs and aptamer; while work is underway to bring drugs in the miRNA and saRNA classes to market. This ongoing development focuses on studying safety profiles and implementing potential modifications to minimize side effects [48]. The modifications of the CaP-siRNA used in this study are intended to minimize its toxicity and develop inappropriate immunostimulation. This seems to be successfully reached, since in the model proposed—even at high concentrations of CaP-siRNA—inappropriate immunostimulation is not detected. As reported in the literature, exogenous RNAs can activate immune responses by interacting with pattern recognition receptors (PRRs) such as TLRs [49]. While in certain cases, such as in vaccine development or cancer immunotherapy, this immune activation may be deliberately utilized to enhance therapeutic effects, in most other treatments, such immunostimulatory effects are unwanted. Immune-related and dose-limiting toxicities are key factors contributing to the failure of numerous RNA drugs in clinical trials. Consequently, the administration of pharmacological RNA agents may induce immune responses or cytokine release syndrome, depending on the dosage and characteristics of the RNA molecules, such as size and sequence [16]. The possible release of cytokines following siRNA treatment has already been documented in the literature. Sioud et al. have found that both single- and double-stranded siRNAs (whether in the sense or antisense strands) could stimulate the release of IL-6 and TNF-α in adherent PBMCs [50]. Notably, only specific siRNA sequences triggered these inflammatory responses, indicating that the immunostimulatory effects of siRNA are influenced by sequence specificity. Furthermore, the activation of TNF-α, IL-6, and interferon-α (IFN-α) was inhibited by chloroquine, suggesting that this immune response was likely mediated through endosomal TLRs; with TRL3 and TLR8 playing a prominent role [50,51]. PRRs often trigger immune responses upon recognizing specific nucleotide sequences, such as RNA rich in guanine-uracil or adenine-uracil motifs, which are known to activate TLR7 and TLR8 receptors; as well as siRNA sequences rich in uracil [50,52]. The results presented in this study demonstrate that the release of the proinflammatory cytokines IL-8, IFN-γ and IL-6 did not show any differences compared to the control. The only significant modulation is the reduction in the release of TNF-*α* induced by 400 nM CaP-siRNA compared both to CaP NPs and CaP-scramble. This evidence reveals not only the efficiency of the modification made on the CaP-siRNA but also the encapsulation in CaP NPs to avoid inappropriate immune response.

Regarding toxicity pathways modulation, the results showed in some cases high variability, but these were not sex-related. Differences in the response indicate different sensitivity in the population, which may predispose some people to potential adverse reactions. Nevertheless, due to the limited sample in this study, further analyses with a larger cohort are required to assess this point more thoroughly. Overall, gene expression data obtained following the treatment with CaP-siRNA did not show a particular trend of modulation; and in many cases, the effects are only significant for a specific concentration. Moreover, no clear difference in behavior is evident between CaP-siRNA and CaP-scramble in most cases. Only BBC3 showed this statistically significant difference, but only at the highest concentration, which deserves further investigation. It is also interesting to note that the increase of the expression of most of the genes is detected only at the higher concentration tested with the CaP-scramble. In some cases, the response to the CaP-scramble is even higher than the one of the positive controls, indicating that the siRNA molecule could induce toxicity by modulating genes involved in ER stress and oxidative stress, as shown by the increase of SOD1 and DDIT3 expression. The lack of dose response or effect observed only at pharmacological doses of siRNA can be interpreted in several ways. Saturation of the target pathway where, at higher doses, the siRNA may saturate its off-target, leading to a plateau or loss of the effect due to overloading cellular processing mechanisms, such as RISC (RNA-induced silencing complex). The effect at pharmacological doses might be due to off-target interactions that are diminished at higher doses, where other cellular pathways or compensatory mechanisms become activated. Feedback mechanisms where higher doses may trigger negative feedback loops or desensitization of pathways, may reduce the observed effect. Differences in delivery efficiency, where the delivery might be more efficient at pharmacological doses than at higher doses, may lead to a decrease in the observed effect at elevated concentrations. Finally, the pharmacokinetics of siRNA uptake and degradation could vary with concentration, leading to changes in its bioavailability and effectiveness. As the genes evaluated in this study are associated with well-known toxicity pathways and some changes are observed with the administration of CaP-siRNA, additional studies are needed to better define the activation of the specific pathways; e.g., analysis of the activation or inhibition of downstream pathways to understand dose-dependent effects, changes at the protein levels and apical endpoints. Extended periods of analysis paired with chronic exposure may further elucidate this aspect.

## 5. Conclusions

The present work describes an in vitro approach developed to screen RNA-drugs for potential immunotoxicity and induction of key gene expression associated with toxicity pathways as a preliminary step in the preclinical safety evaluation of RNA-based drugs. As a proof of principle, the CaP-siRNA, developed within the Italian National Center for Gene Therapy and Drugs based on RNA Technology, was used. The results showed that the drug developed with its loading in CaP NPs and modification of the siRNA are not cytotoxic at the tested concentrations. Moreover, the investigation of cytokine release showed that this type of drug does not trigger inappropriate immunostimulation. Specifically, the RNA-drug does not induce the release of TNF-α, IL-8, IL-6, and IFN-γ. Minimal changes in activity were detected in the analysis of toxicity pathway genes, with no clear trend of induction.

Despite these promising findings, one of the major limitations of this approach is the lack of validated positive controls for RNA drugs (siRNA), as no universally recognized positive control siRNA currently exists. Additionally, the in vitro model itself is characterized by significant variability among donors, which can influence the reproducibility of results.

This study highlights a novel application of PBMCs that may be suitable for the safety evaluation of RNA-drugs, albeit with the understanding that these limitations must be considered in the interpretation of the data.

## 6. Patents

CaP: WO2016/102576 and siRNA: WO2017/141157.

## Figures and Tables

**Table 1:** Genes selected for the analysis of the modulation of the toxicity pathways.

Genes	Pathway	Reference
*mTOR*	Autophagy	[37]
*ULK1*	Autophagy	[38]
*ATF4*	Endoplasmic reticulum stress	[39]
*BBC3*	Endoplasmic reticulum stress	[40]
*DDIT3*	Endoplasmic reticulum stress	[41]
*ATF5*	Mitochondrial stress	[42]
*SOD1*	Oxidative stress	[43]

**Table 2:** Physico-chemical characterization of siRNA-loaded CaP NPs (PDI: Polydispersity index).

Sample	Z-Average (nm)	PDI	ζ-Potential (mV)	CaP NPs Concentration (mg/mL)	siRNA Loading (wt.%)
CaP NPs	65 ± 6	0.30 ± 0.03	−34 ± 3	0.6 ± 0.2	-
CaP-siRNA	40 ± 5	0.31 ± 0.02	−35 ± 4	0.6 ± 0.1	2.7 ± 0.3
CaP-scramble	50 ± 5	0.32 ± 0.03	−33 ± 5	0.6 ± 0.1	1.8 ± 0.2

## Data Availability

Data are contained within the article.

## References

[1] Roberts T.C., Langer R., Wood M.J.A. (2020). Advances in Oligonucleotide Drug Delivery. Nat. Rev. Drug Discov..

[2] Lam J.K.W., Chow M.Y.T., Zhang Y., Leung S.W.S. (2015). SiRNA Versus MiRNA as Therapeutics for Gene Silencing. Mol. Ther. Nucleic Acids.

[3] Crooke S.T., Witztum J.L., Bennett C.F., Baker B.F. (2018). RNA-Targeted Therapeutics. Cell Metab..

[4] Arechavala-Gomeza V., Garanto A. (2022). Antisense RNA Therapeutics: A Brief Overview. Antisense RNA Design, Delivery, and Analysis.

[5] Moreno-Montañés J., Sádaba B., Ruz V., Gómez-Guiu A., Zarranz J., González M.V., Pañeda C., Jimenez A.I. (2014). Phase I Clinical Trial of SYL040012, a Small Interfering RNA Targeting β-Adrenergic Receptor 2, for Lowering Intraocular Pressure. Mol. Ther..

[6] Benitez-Del-Castillo J.M., Moreno-Montañés J., Jiménez-Alfaro I., Muñoz-Negrete F.J., Turman K., Palumaa K., Sádaba B., González M.V., Ruz V., Vargas B. (2016). Safety and Efficacy Clinical Trials for SYL1001, a Novel Short Interfering RNA for the Treatment of Dry Eye Disease. Investig. Opthalmology Vis. Sci..

[7] Nguyen Q.D., Schachar R.A., Nduaka C.I., Sperling M., Klamerus K.J., Chi-Burris K., Yan E., Paggiarino D.A., Rosenblatt I., Aitchison R. (2012). Evaluation of the SiRNA PF-04523655 versus Ranibizumab for the Treatment of Neovascular Age-Related Macular Degeneration (MONET Study). Ophthalmology.

[8] Adams D., Gonzalez-Duarte A., O’Riordan W.D., Yang C.-C., Ueda M., Kristen A.V., Tournev I., Schmidt H.H., Coelho T., Berk J.L. (2018). Patisiran, an RNAi Therapeutic, for Hereditary Transthyretin Amyloidosis. N. Engl. J. Med..

[9] Raal F.J., Kallend D., Ray K.K., Turner T., Koenig W., Wright R.S., Wijngaard P.L.J., Curcio D., Jaros M.J., Leiter L.A. (2020). Inclisiran for the Treatment of Heterozygous Familial Hypercholesterolemia. N. Engl. J. Med..

[10] Ray K.K., Wright R.S., Kallend D., Koenig W., Leiter L.A., Raal F.J., Bisch J.A., Richardson T., Jaros M., Wijngaard P.L.J. (2020). Two Phase 3 Trials of Inclisiran in Patients with Elevated LDL Cholesterol. N. Engl. J. Med..

[11] Tanna S., Doshi G., Godad A. (2024). SiRNA as Potential Therapeutic Strategy for Hypertension. Eur. J. Pharmacol..

[12] Desai A.S., Webb D.J., Taubel J., Casey S., Cheng Y., Robbie G.J., Foster D., Huang S.A., Rhyee S., Sweetser M.T. (2023). Zilebesiran, an RNA Interference Therapeutic Agent for Hypertension. N. Engl. J. Med..

[13] Zhu Y., Zhu L., Wang X., Jin H. (2022). RNA-Based Therapeutics: An Overview and Prospectus. Cell Death Dis..

[14] Paunovska K., Loughrey D., Dahlman J.E. (2022). Drug Delivery Systems for RNA Therapeutics. Nat. Rev. Genet..

[15] Alshaer W., Zureigat H., Al Karaki A., Al-Kadash A., Gharaibeh L., Hatmal M.M., Aljabali A.A.A., Awidi A. (2021). SiRNA: Mechanism of Action, Challenges, and Therapeutic Approaches. Eur. J. Pharmacol..

[16] Yu A.-M., Choi Y.H., Tu M.-J. (2020). RNA Drugs and RNA Targets for Small Molecules: Principles, Progress, and Challenges. Pharmacol. Rev..

[17] Andersson P. (2022). Preclinical Safety Assessment of Therapeutic Oligonucleotides. Antisense RNA Design, Delivery, and Analysis.

[18] Beg M.S., Brenner A.J., Sachdev J., Borad M., Kang Y.-K., Stoudemire J., Smith S., Bader A.G., Kim S., Hong D.S. (2017). Phase I Study of MRX34, a Liposomal MiR-34a Mimic, Administered Twice Weekly in Patients with Advanced Solid Tumors. Investig. New Drugs.

[19] Anderson B.R., Muramatsu H., Nallagatla S.R., Bevilacqua P.C., Sansing L.H., Weissman D., Karikó K. (2010). Incorporation of Pseudouridine into MRNA Enhances Translation by Diminishing PKR Activation. Nucleic Acids Res..

[20] Tanji H., Ohto U., Shibata T., Taoka M., Yamauchi Y., Isobe T., Miyake K., Shimizu T. (2015). Toll-like Receptor 8 Senses Degradation Products of Single-Stranded RNA. Nat. Struct. Mol. Biol..

[21] Janas M.M., Schlegel M.K., Harbison C.E., Yilmaz V.O., Jiang Y., Parmar R., Zlatev I., Castoreno A., Xu H., Shulga-Morskaya S. (2018). Selection of GalNAc-Conjugated SiRNAs with Limited off-Target-Driven Rat Hepatotoxicity. Nat. Commun..

[22] Yan Y., Liu X.-Y., Lu A., Wang X.-Y., Jiang L.-X., Wang J.-C. (2022). Non-Viral Vectors for RNA Delivery. J. Control. Release.

[23] Szebeni J., Simberg D., González-Fernández Á., Barenholz Y., Dobrovolskaia M.A. (2018). Roadmap and Strategy for Overcoming Infusion Reactions to Nanomedicines. Nat. Nanotechnol..

[24] Bitounis D., Jacquinet E., Rogers M.A., Amiji M.M. (2024). Strategies to Reduce the Risks of MRNA Drug and Vaccine Toxicity. Nat. Rev. Drug Discov..

[25] Priori S.G., Napolitano C., Tiso N., Memmi M., Vignati G., Bloise R., Sorrentino V., Danieli G.A. (2001). Mutations in the Cardiac Ryanodine Receptor Gene (HRyR2) Underlie Catecholaminergic Polymorphic Ventricular Tachycardia. Circulation.

[26] Abdullah N.M., Ali A. (2024). RYR2 Receptor Gene Mutation Associated with Catecholaminergic Polymorphic Ventricular Tachycardia in Children: A Case Report & Literature Review. Transl. Pediatr..

[27] Bers D.M. (2002). Cardiac Excitation–Contraction Coupling. Nature.

[28] Liu N., Colombi B., Memmi M., Zissimopoulos S., Rizzi N., Negri S., Imbriani M., Napolitano C., Lai F.A., Priori S.G. (2006). Arrhythmogenesis in Catecholaminergic Polymorphic Ventricular Tachycardia. Circ. Res..

[29] Bongianino R., Denegri M., Mazzanti A., Lodola F., Vollero A., Boncompagni S., Fasciano S., Rizzo G., Mangione D., Barbaro S. (2017). Allele-Specific Silencing of Mutant MRNA Rescues Ultrastructural and Arrhythmic Phenotype in Mice Carriers of the R4496C Mutation in the Ryanodine Receptor Gene (RYR2). Circ. Res..

[30] Di Mauro V., Iafisco M., Salvarani N., Vacchiano M., Carullo P., Ramírez-Rodríguez G.B., Patrício T., Tampieri A., Miragoli M., Catalucci D. (2016). Bioinspired Negatively Charged Calcium Phosphate Nanocarriers for Cardiac Delivery of MicroRNAs. Nanomedicine.

[31] Miragoli M., Ceriotti P., Iafisco M., Vacchiano M., Salvarani N., Alogna A., Carullo P., Ramirez-Rodríguez G.B., Patrício T., Esposti L.D. (2018). Inhalation of Peptide-Loaded Nanoparticles Improves Heart Failure. Sci. Transl. Med..

[32] Modica J., Di Mauro V., Barandalla-Sobrados M., Chavez S.E.P., Carullo P., Nemska S., Anselmo A., Condorelli G., Iafisco M., Miragoli M. (2021). Nano-MiR-133a Replacement Therapy Blunts Pressure Overload–Induced Heart Failure. Circulation.

[33] Alogna A., Berboth L., Faragli A., Ötvös J., lo Muzio F.P., di Mauro V., Modica J., Quarta E., Semmler L., Deißler P.M. (2024). Lung-to-Heart Nano-in-Micro Peptide Promotes Cardiac Recovery in a Pig Model of Chronic Heart Failure. J. Am. Coll. Cardiol..

[34] Savi M., Rossi S., Bocchi L., Gennaccaro L., Cacciani F., Perotti A., Amidani D., Alinovi R., Goldoni M., Aliatis I. (2014). Titanium Dioxide Nanoparticles Promote Arrhythmias via a Direct Interaction with Rat Cardiac Tissue. Part. Fibre Toxicol..

[35] Mills N.L., Donaldson K., Hadoke P.W., Boon N.A., MacNee W., Cassee F.R., Sandström T., Blomberg A., Newby D.E. (2009). Adverse Cardiovascular Effects of Air Pollution. Nat. Clin. Pract. Cardiovasc. Med..

[36] Iafisco M., Alogna A., Miragoli M., Catalucci D. (2019). Cardiovascular Nanomedicine: The Route Ahead. Nanomedicine.

[37] Yu L., McPhee C.K., Zheng L., Mardones G.A., Rong Y., Peng J., Mi N., Zhao Y., Liu Z., Wan F. (2010). Termination of Autophagy and Reformation of Lysosomes Regulated by MTOR. Nature.

[38] Qiu X., Zheng L., Liu X., Hong D., He M., Tang Z., Tian C., Tan G., Hwang S., Shi Z. (2021). ULK1 Inhibition as a Targeted Therapeutic Strategy for Psoriasis by Regulating Keratinocytes and Their Crosstalk With Neutrophils. Front. Immunol..

[39] Verfaillie T., Salazar M., Velasco G., Agostinis P. (2010). Linking ER Stress to Autophagy: Potential Implications for Cancer Therapy. Int. J. Cell Biol..

[40] Corazzari M., Gagliardi M., Fimia G.M., Piacentini M. (2017). Endoplasmic Reticulum Stress, Unfolded Protein Response, and Cancer Cell Fate. Front. Oncol..

[41] Zhao Q. (2002). A Mitochondrial Specific Stress Response in Mammalian Cells. EMBO J..

[42] Zhou D., Palam L.R., Jiang L., Narasimhan J., Staschke K.A., Wek R.C. (2008). Phosphorylation of EIF2 Directs ATF5 Translational Control in Response to Diverse Stress Conditions. J. Biol. Chem..

[43] Glasauer A., Sena L.A., Diebold L.P., Mazar A.P., Chandel N.S. (2014). Targeting SOD1 Reduces Experimental Non–Small-Cell Lung Cancer. J. Clin. Investig..

[44] Xu X., Li Z., Zhao X., Keen L., Kong X. (2016). Calcium Phosphate Nanoparticles-Based Systems for SiRNA Delivery. Regen. Biomater..

[45] Degli Esposti L., Carella F., Adamiano A., Tampieri A., Iafisco M. (2018). Calcium Phosphate-Based Nanosystems for Advanced Targeted Nanomedicine. Drug Dev. Ind. Pharm..

[46] Lee Y., Jeong M., Park J., Jung H., Lee H. (2023). Immunogenicity of Lipid Nanoparticles and Its Impact on the Efficacy of MRNA Vaccines and Therapeutics. Exp. Mol. Med..

[47] Najahi-Missaoui W., Arnold R.D., Cummings B.S. (2020). Safe Nanoparticles: Are We There Yet?. Int. J. Mol. Sci..

[48] Qiu L., Jing Q., Li Y., Han J. (2023). RNA Modification: Mechanisms and Therapeutic Targets. Mol. Biomed..

[49] Pauls E., Senserrich J., Bofill M., Clotet B., Esté J.A. (2006). Induction of Interleukins IL-6 and IL-8 by SiRNA. Clin. Exp. Immunol..

[50] Sioud M. (2005). Induction of Inflammatory Cytokines and Interferon Responses by Double-Stranded and Single-Stranded SiRNAs Is Sequence-Dependent and Requires Endosomal Localization. J. Mol. Biol..

[51] Meng Z., Lu M. (2017). RNA Interference-Induced Innate Immunity, Off-Target Effect, or Immune Adjuvant?. Front. Immunol..

[52] Robbins M., Judge A., MacLachlan I. (2009). SiRNA and Innate Immunity. Oligonucleotides.

